# Supplementary material for: In vitro reconstitution of an efficient nucleotide excision repair system using mesophilic enzymes from Deinococcus radiodurans
Source: Commun Biol. 2022 Feb 11;5:127. doi: 10.1038/s42003-022-03064-x (PMC8837605; doi:10.1038/s42003-022-03064-x)
Supplement: Supplementary file 2 — Supplementary Information [file 42003_2022_3064_MOESM2_ESM.pdf]

In vitro reconstitution of an efficient nucleotide excision repair system using mesophilic enzymes from *Deinococcus radiodurans*

Anna Seck[1,2], Salvatore De Bonis[1], Christine Saint-Pierre[2], Didier Gasparutto[2], Jean-Luc Ravanat[2*] and Joanna Timmins[1*].

[1]Univ. Grenoble Alpes, CEA, CNRS, IBS, F-38000 Grenoble, France.

[2]Univ. Grenoble Alpes, CEA, CNRS, SyMMES-UMR 5819, F-38000 Grenoble, France.

*Corresponding authors: Joanna Timmins (Joanna.timmins@ibs.fr) and Jean-Luc Ravanat (jean-luc.ravanat@cea.fr)

Supplementary Data

Supplementary Figure 1

Supplementary Figure 2

Supplementary Figure 3

Supplementary Figure 4

Supplementary Figure 5

Supplementary Figure 6

Supplementary Table 1

Supplementary Table 2

Supplementary Table 3

Supplementary Table 4

Supplementary Table 5

Supplementary Data 1 (separate Excel file corresponding to source data)

Supplementary Figure 1

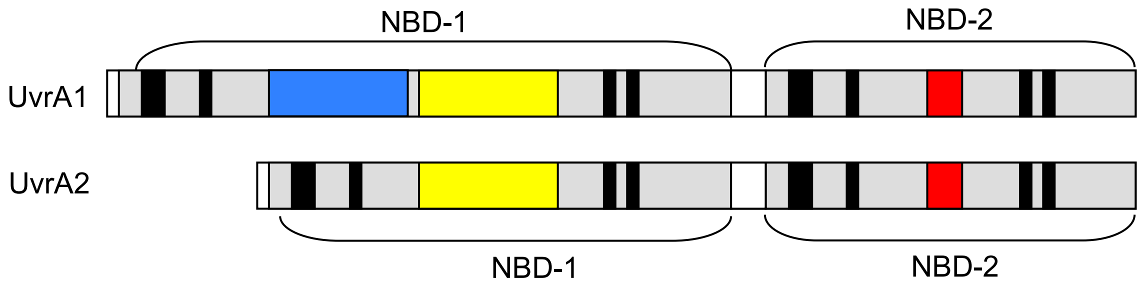

Supplementary Figure 1. Schematic diagram illustrating the domain composition of class I (UvrA1) and class II (UvrA2) UvrA proteins. Both UvrAs are composed of two tandem nucleotide-binding domains (NBDs) bearing Walker A and Walker B motifs, a Q-loop and a signature motif (black rectangles) that together form the two composite ATP binding sites at the interface between the two NBDs. In addition, UvrAs contain a conserved N-terminal insertion domain (yellow) and a C-terminal zinc-finger motif (red), both of which are involved in DNA binding. The N-terminal NBD of UvrA1 bears an additional zinc-binding domain (blue), which is missing in UvrA2 proteins, that has been reported to be the UvrB interacting domain of UvrA.

Supplementary Figure 2

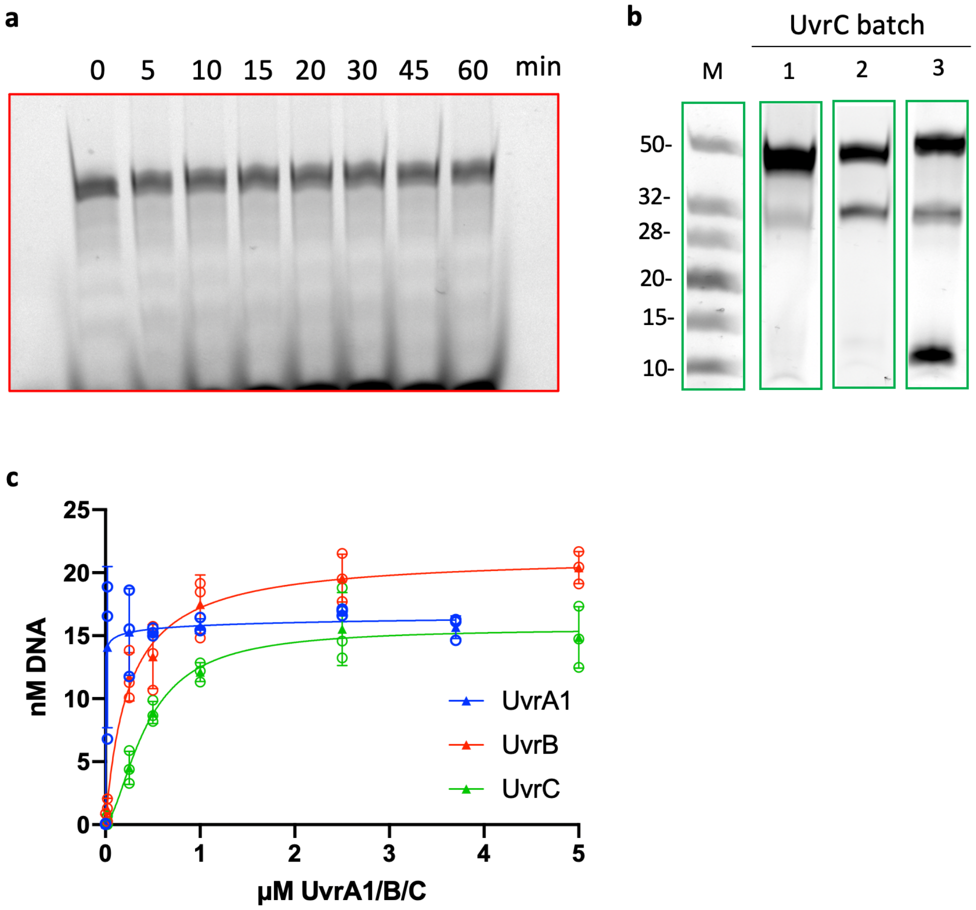

Supplementary Figure 2. (a) TBE-polyacrylamide urea gel analysis of a timecourse experiment using drUvrABC and the control substrate, seq1-ATTO, with an unconjugated thymine in position 26 (see Supplementary Tables 1 and 2). Reactions were performed for up to 60 minutes at 37°C using 25 nM 25 nM seq1-ATTO substrate and 1 μM drUvrA1, 0.5 μM drUvrB and 2 μM drUvrC in the presence of 2.5 mM Mg^{2+} and 2.5 mM ATP. The gel was visualized with the red filter to detect ATTO633-labelled bands. No incision activity could be detected. (b) TBE-polyacrylamide urea gel analyses of the drUvrABC incision activity using different batches of purified drUvrC protein. Reactions were performed for 1 hour at 37°C using 25 nM F26-seq1 substrate, 1 μM drUvrA1, 0.5 μM drUvrB and 2 μM drUvrC in the presence of 10 mM Mg^{2+} and 4 mM ATP. Lane 1: incision by a partially cleaved drUvrC still containing its N-terminal His-tag and some residual nucleic acid contamination. Lane 2: incision by drUvrC purified on Ni-NTA resin which was visibly stripped of its nickel after elution of drUvrC. Lane 3: incision by drUvrC after optimization of the purification protocol. The gels were visualized with the green filter to detect fluorescein-labelled bands. (c) Dual incision activity by drUvrABC as a function of protein concentration. Reactions were performed at 37°C for 45 min using

25 nM F26-seq1 substrate and either 0-3.7 µM drUvrA1, 0.5 µM drUvrB and 2 µM drUvrC (blue line), 1 µM drUvrA1, 0-5 µM UvrB and 2 µM UvrC (red line) or 1 µM drUvrA1, 0.5 µM UvrB and 0-5 µM UvrC (green line). All reactions contained 2.5 mM $MgCl_2$ and were started by addition of 2.5 mM ATP. The graph presents the mean amount of 12 mer product (nM) released after 45 minutes (filled tringles) and standard deviation of three individual replicates shown as open circles.

Supplementary Figure 3

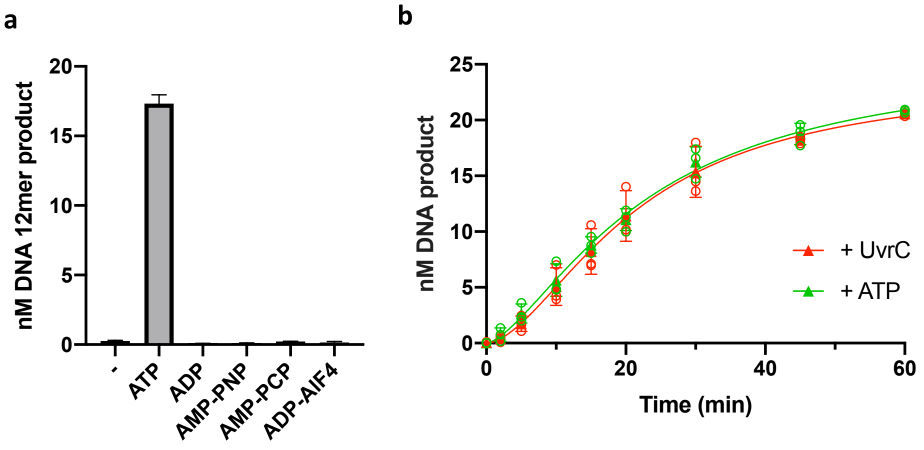

Supplementary Figure 3. (a) Dual incision activity by drUvrABC in the presence of either ATP, ADP or non-hydrolysable analogues of ATP (AMP-PNP, AMP-PCP or ADP-AlF_4^-). Reactions were performed at 37°C for 45 min using 25 nM F26-seq1 substrate, 1 µM drUvrA1, 0.5 µM drUvrB and 2 µM drUvrC. All reactions contained 2.5 mM $MgCl_2$ and were started by addition of 2.5 mM nucleotide. Histograms correspond to the mean amount of 12 mer product (nM) released after 45 minutes and standard deviation of three individual replicates. (b) Kinetics of the dual incision reaction performed by drUvrABC. Reactions were performed at 37°C using 25 nM F26-seq1 substrate, 1 µM drUvrA1, 0.5 µM drUvrB and 2 µM drUvrC, 2.5 mM $MgCl_2$ and 2.5 mM ATP, and were started either by addition of the ATP (green) or by addition of drUvrC (red) after a pre-incubation of drUvrA1 and drUvrB with the DNA substrate and ATP for 10 min. The graph presents the mean amount of 12 mer product (nM) released at each time point (filled triangles) and standard deviation of three individual replicates shown as open circles.

Supplementary Figure 4

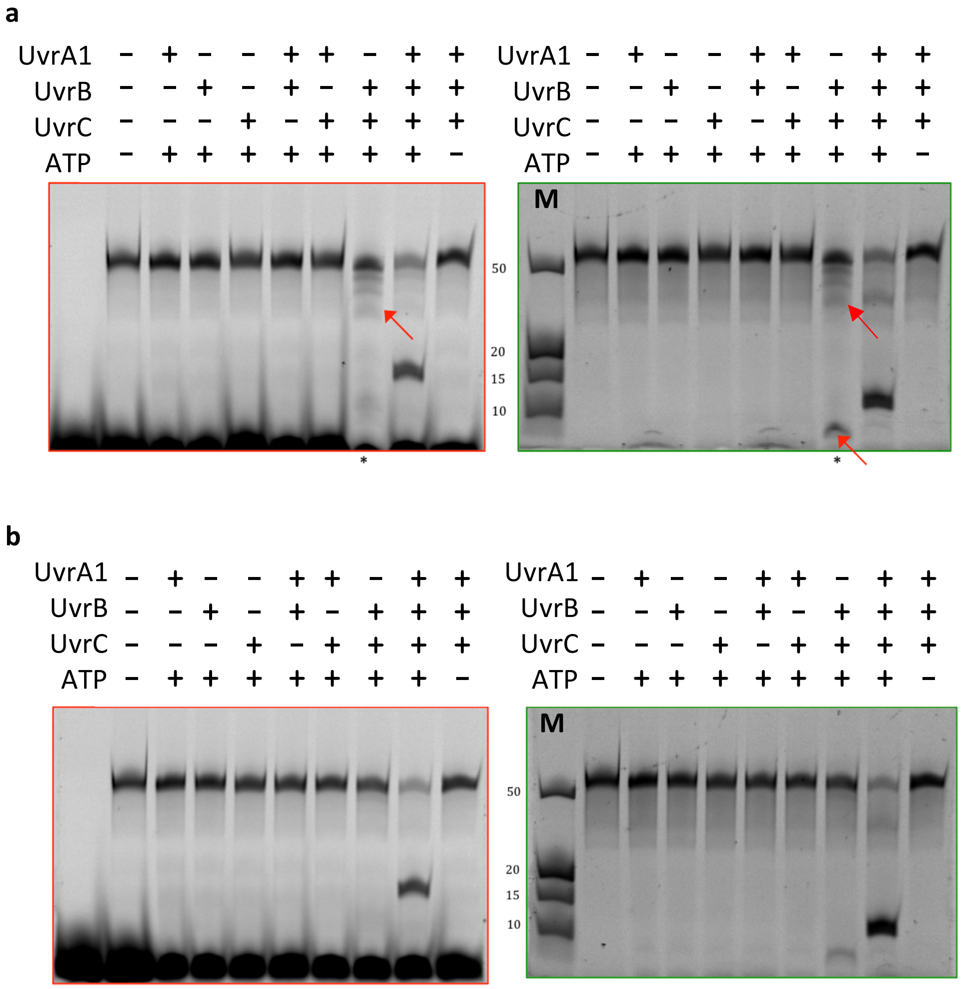

Supplementary Figure 4. TBE-polyacrylamide urea gel analysis of the drUvrABC incision activity in the presence of Mn^{2+} or Mn^{2+} supplemented with Fe^{3+}. Reactions were performed for 45 minutes at 37°C using 25 nM F26-seq1 substrate and different combinations of drUvrA1 (1µM), drUvrB (0.5µM) and drUvrC (2µM) in the presence of 2.5 mM Mn^{2+} (a) or 2.5 mM Mn^{2+} and 0.25 mM Fe^{3+} (b). Reactions were started by addition of 2.5 mM ATP. Left gel: gel visualized with the red filter to detect ATTO633-labelled bands. Right gel: gel visualized with the green filter to detect fluorescein-labelled bands. Left lane: molecular weight marker composed of fluorescein-labelled oligonucleotides ranging from 10 to 50 bp. Red arrows indicate non-specific incision products obtained with drUvrB and drUvrC in the presence of Mn^{2+}.

Supplementary Figure 5

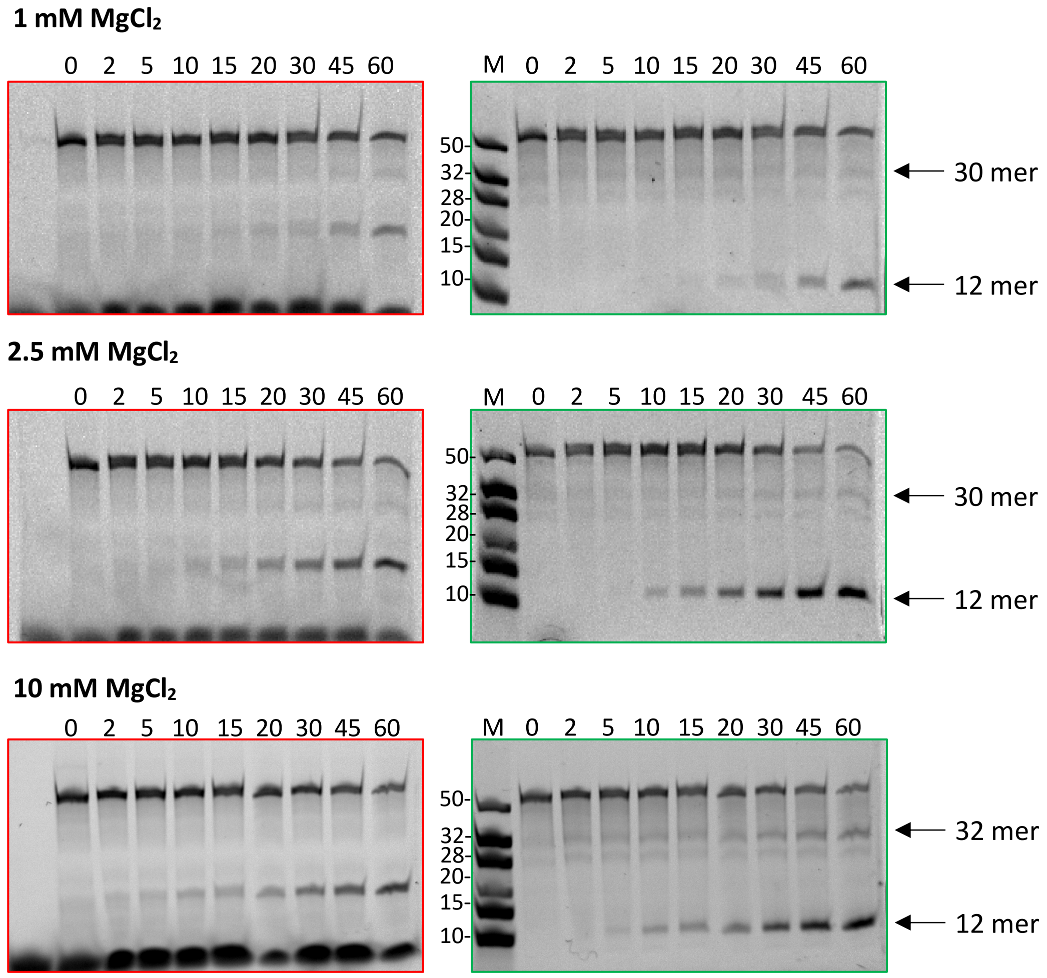

Supplementary Figure 5. Effects of $MgCl_2$ concentration on the dual incision activity of drUvrABC. TBE-polyacrylamide gel analysis of timecourse experiments performed at 37°C for 1 hour using 25 nM F26-seq1 substrate, 1 µM drUvrA1, 0.5 µM drUvrB and 2 µM drUvrC, and either 1 mM (top), 2.5 mM (middle) or 10 mM (bottom) $MgCl_2$. Left gel: gel visualized with the red filter to detect ATTO633-labelled bands. Right gel: gel visualized with the green filter to detect fluorescein-labelled bands. Left lane: molecular weight marker composed of fluorescein-labelled oligonucleotides ranging from 10 to 50 bp. Arrows indicate final and intermediate DNA products observed in each case. The 30 mer fragment is visible with both the red and the green filter, whereas the 32 mer band is only green-labelled.

Supplementary Figure 6

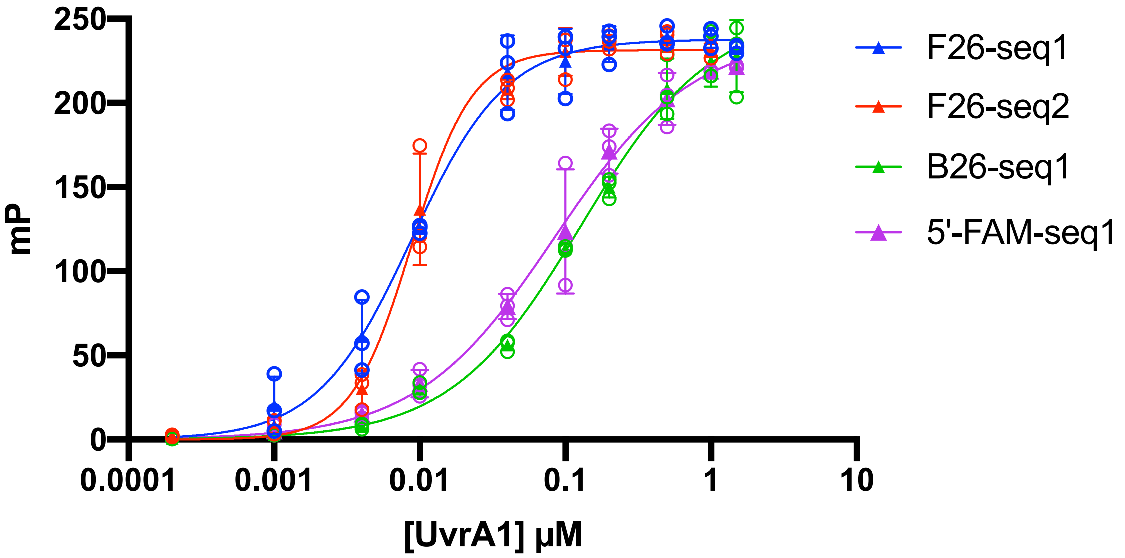

Supplementary Figure 6. Binding curves derived from the fluorescence polarization measurements of drUvrA1 binding to different 50mer dsDNA substrates (Supplementary Table 2): F26-seq1 (blue), F26-seq2 (red), B26-seq1 (green) and 5'-FAM-seq1 (purple). The graph presents the mean (filled triangles) and standard deviation of at least three independent measurements shown as open circles, and the best fits.

Supplementary Table 1. Observed rate constants (K_{obs}) of product release by *D. radiodurans* UvrABC

drUvrABC concentration (µM)	K_{obs} (min^{-1} x 10^{-3}) F26-seq1	K_{obs} (min^{-1} x 10^{-3}) F26-seq2	K_{obs} (min^{-1} x 10^{-3}) B26-seq1*
UvrA1=1; UvrB=0.5; UvrC=2	31.3 ± 3.5	73.6 ± 7.4	33.5 ± 2.3
UvrA1=0.5; UvrB=0.25; UvrC=1	24.7 ± 4.2	72.6 ± 3.0	8.4 ± 0.2
UvrA1=0.25; UvrB=0.125; UvrC=0.5	26.7 ± 2.6	70.6 ± 4.4	3.5 ± 0.4
UvrA1=0.1; UvrB=0.05; UvrC=0.2	37.1 ± 3.2	61.1 ± 3.6	ND

* Rate constants determined using 5'-FAM-labelled 18mer DNA fragment data.

Supplementary Table 2. Oligonucleotides used to prepare DNA substrates used in this study

Name	Sequence	Modification(s)
5'-ATTO633-seq1	5'- X - GAC TAC GTA CTG TTA CGG CTC CAT CTC TAC CGC AAT CAG GCC AGA TCT GC -3'	X = ATTO633
5'-FAM-seq1	5'- X - GAC TAC GTA CTG TTA CGG CTC CAT CTC TAC CGC AAT CAG GCC AGA TCT GC -3'	X = FAM
5'-ATTO633-seq1	5'- X - GAC TAC GTA CTG TTA CGG CTC CAT CTC TAC CGC AAT CAG GCC AGA TCT GC -3'	X = ATTO633
5'-ATTO633-F26-seq1	5'- X - GAC TAC GTA CTG TTA CGG CTC CAT CYC TAC CGC AAT CAG GCC AGA TCT GC -3'	X = ATTO633<br>Y = Fluo-dT
5'-ATTO633-F26-seq2	5'- X - GTT AGC GAA CGA TAC CTT CAG TAG AYC AAG TCC TAG CTG ACC GGT TCG GC -3'	X = ATTO633<br>Y = Fluo-dT
Rev-seq1	5'- GCA GAT CTG GCC TGA TTG CGG TAG AGA TGG AGC CGT AAC AGT ACG TAG TC -3'	
Rev-seq2	5'- GCC GAA CCG GTC AGC TAG GAC TTG A T CTA CTG AAG GTA TCG TTC GCT AAC -3'	
5'-FAM-B26-seq1	5'- X - GAC TAC GTA CTG TTA CGG CTC CAT CYC TAC CGC AAT CAG GCC AGA TCT GC -3'	X = FAM<br>Y = Biotin-dT

Supplementary Table 3. DNA substrates used in this study

Name	Oligo 1	Oligo 2
F26-seq1	5'-ATTO633-F26-seq1	Rev-seq1
F26-seq2	5'-ATTO633-F26-seq2	Rev-seq2
B26-seq1	5'-FAM-B26-seq1	Rev-seq1
seq1-FAM	5'-FAM-seq1	Rev-seq1
Seq1-ATTO	5'-ATTO633-seq1	Rev-seq1
B26-strep-seq1	5'-FAM-B26-seq1 + strep	Rev-seq1

Supplementary Table 4: Expected and measured masses of DNA fragments after processing of F26-seq2 by *D. radiodurans* UvrABC.

Oligonucleotide	Sequence	Expected mass (Da)	Measured mass (Da)
5'-ATTO633-F26-seq2 (substrate)	5'- **X**GTT AGC GAA CGA TAC CTT CAG TAG A**FdT**C AAG TCC TAG CTG ACC GGT TCG GC -3'	**16 609.0**	**16 602.1**
Rev-seq2 (substrate)	5'- GCC GAA CCG GTC AGC TAG GAC TTG A T CTA CTG AAG GTA TCG TTC GCT AAC -3'	**15 386.0**	**15 380.7**
12mer (product)	5'- **p**CAGTAGA**FdT**CAAG -3'	**4 269.4**	**4 271.8**
18mer (product)	5'- **X**GTTAGCGAACGATACCTT -3'	**6 210.6**	**6 212.2**
20mer (product)	5'- **p**TCCTAGCTGACCGGTTCGGC -3'	**6 165.0**	**6 166.4**

X= ATTO633; FdT= fluorescein-conjugated thymine; p=5' phosphate group

Supplementary Table 5: Expected and measured masses of DNA fragments after processing of B26-seq1 by *D. radiodurans* UvrABC.

Oligonucleotide	Sequence	Expected mass (Da)	Measured mass (Da)
5'-FAM-B26-seq1 (substrate)	5'- **X**GAC TAC GTA CTG TTA CGG CTC CAT C**BdT**C TAC CGC AAT CAG GCC AGA TCT GC -3'	**16 160.0**	**16 159.0**
Rev-seq1 (substrate)	5'- GCA GAT CTG GCC TGA TTG CGG TAG AGA TGG AGC CGT AAC AGT ACG TAG TC -3'	**15 531.0**	**15 531.8**
12mer (product)	5'- **p**CTCCATC**BdT**CTAC -3'	**3 976.4**	**3 975.6**
18mer (product)	5'- **X**GACTACGTACTGTTACGG -3'	**6 052.6**	**6 054.7**
20mer (product)	5'- **p**CGCAATCAGGCCAGATCTGC -3'	**6 167.0**	**6 168.0**

X= ATTO633; FdT= fluorescein-conjugated thymine; p=5' phosphate group
